# Experiences of Women with Disabilities in Accessing Maternal Healthcare Services: A South African Case Study

**DOI:** 10.3390/ijerph20216966

**Published:** 2023-10-24

**Authors:** Doreen Mheta, Maureen Nokuthula Sibiya, Pauline Busisiwe Nkosi

**Affiliations:** 1 Faculty of Health Sciences, Durban University of Technology, Durban 4000, South Africa; paulinen1@dut.ac.za; 2 Division of Research, Innovation and Engagement, Mangosuthu University of Technology, Durban 4031, South Africa; sibiya.nokuthula@mut.ac.za

**Keywords:** disability, maternal health, maternal healthcare services, pregnancy, access

## Abstract

Access to maternal healthcare services is a challenge in most low- and middle-income countries. South Africa is one of the countries striving to improve the accessibility of maternal healthcare services. Although South Africa has put some interventions in place to improve the accessibility of maternal healthcare services, vulnerable women including women with disabilities are still facing numerous challenges when trying to access these services. The aim of this study was to explore the experiences of women with disabilities in the province of KwaZulu-Natal in South Africa in accessing public maternal healthcare services. The objectives of this study were to describe the experiences of women with disabilities in accessing maternal healthcare services during pregnancy, childbirth and post-partum care; explore the inhibitors of access to maternal healthcare services for women with disabilities; and explore the facilitators of access to maternal healthcare services for women with disabilities. Twelve women with disabilities (four with physical impairments, four with hearing impairments and four with visual impairments) were interviewed for this study. Data were transcribed verbatim and analysed utilising the Framework of Assessing Access to Maternal Healthcare Services by Peters et al., 2008. Our study found that narrow passages and information in inaccessible formats were a challenge for women with visual impairments. Women with hearing impairments faced communication difficulties due to the lack of sign language interpreters in most facilities. Moreover, healthcare professionals displayed unfavourable attitudes toward women with hearing impairments, and these women were often overlooked when seeking help. The women with physical impairments encountered inaccessible buildings, narrow passages, small consultation rooms and equipment that is not adjustable, such as beds and scales.

## 1. Introduction

Disability is an important public health issue in low-, middle- and high-income countries [1,2]. Evidence shows that the healthcare needs of people with disabilities are generally not adequately met due to structural, financial and attitudinal barriers to access [3,4]. Although people with disabilities face challenges in accessing healthcare services, women with disabilities (WWDs) have more challenges compared to their male counterparts. This is because women with disabilities (WWDs) are often associated with higher rates of poverty, lower educational attainment and lower wages compared to disabled men [5,6]. When it comes to access to maternal healthcare services, WWDs are worse off due to the prevalence of the dominant misconception that they are asexual and thus do not require reproductive healthcare services [7]. Pregnancy and motherhood are considered taboo amongst this population [8]. Although the majority of individuals with disabilities live in low- and middle-income nations, research focusing on the maternal healthcare experiences of WWDs is increasing in high-income countries rather than in low- and middle-income nations [9,10].

The government of South Africa enshrined equality in Section 9 of the Bill of Rights [11,12]. Article 3 of Section 9 states that “the state may not unfairly discriminate directly or indirectly against anyone on one or more grounds, including race, gender, sex, pregnancy, marital status, ethnic or social origin, colour, sexual orientation, age, disability, religion, conscience, belief, culture, language and birth” [12]. Non-discrimination of people with disabilities is further emphasised by the South Africa White Paper on the Rights of Persons with Disabilities [13], which has its vision as “South Africa: A free and just society inclusive of all persons with disabilities as equal citizens”. Furthermore, Section 27 of the South African Constitution recognises access to health as a human right [12]. Moreover, the increased public consciousness regarding disability rights has not resulted in a proportional increase in research regarding healthcare accessibility for individuals with disabilities, particularly concerning maternal healthcare services for WWDs.

Although there is strong political commitment on the part of the South African government to address inequalities and discrimination faced by people with disabilities, there is still a gap between policies and their implementation [14]. For instance, the government introduced the free healthcare policy for pregnant women and children under the age of six in 2002 [15], which was then extended to people with disabilities in 2004 [14]. However, a considerable proportion of the exempt groups still pay to utilise services [16]. This has led to healthcare services being difficult to access for a significant portion of the disadvantaged population in South Africa. Due to the prevalent challenges in accessing healthcare services among a considerable proportion of disadvantaged individuals, Vergunst et al. assert that the South African health system is deficient in providing fair access to essential and effective healthcare [17]. Although South Africa frequently experiences barriers to access to healthcare services, there is a lack of in-depth understanding regarding the obstacles faced by people with disabilities when trying to access healthcare services [15].

A few available studies highlight the considerable obstacles that individuals with disabilities encounter when attempting to access healthcare services [2,16]. The situation is notably graver for WWDs than for men with disabilities. While access to healthcare services in general is a challenge, public maternal healthcare services are inadequately equipped to cater to the needs of WWDs [17]. Despite extensive documentation of the factors impeding access to such services for women in general in South Africa, there is limited research elucidating the factors that hinder or facilitate access to maternal healthcare services for WWDs [18,19]. This study therefore aims to explore the experiences of WWDs in the province of KwaZulu-Natal (KZN) in South Africa in accessing public maternal healthcare services and answer the question, what are the perceptions of women with disabilities with regard to access to maternal healthcare?

## 2. Methods

The objective of the study was to describe the experiences of WWDs in accessing maternal healthcare services during pregnancy, childbirth and postpartum care. A qualitative case study research design employing the case study approach was utilised with the underpinning of an interpretive paradigm. The selected research design enabled the researcher to generate in-depth information on the experiences of WWDs. The case study research design was chosen because it allows for a thorough exploration of a specific phenomenon, issue or object within real-life contexts through empirical investigation [18]. Moreover, research suggests that case studies prove valuable in research endeavours that are less developed, especially when it is crucial to investigate the context and situational dynamics [18]. This study focused on maternal healthcare services, and the central topic of discussion was access to these services. It was impossible to investigate the topic of access to maternal healthcare services from the broader context of maternal healthcare services itself. As a result, a case study was the most suitable design as it allowed both the context and the phenomenon to be explored in depth. The study utilised the interpretive paradigm which allowed the researcher to explore the accessibility of maternal healthcare services through the perspectives of the WWDs. Interpretive methodologies focus on comprehending phenomena through an individual’s viewpoint, exploring interactions among individuals and considering the historical and cultural contexts in which people live [19].

This research was conducted in KwaZulu-Natal (KZN), which is one of the nine provinces and has the second largest population in South Africa, located in the southeast of the country [20,21]. The Statistics South Africa Report based on Census 2011 data also indicates that KZN’s provincial disability prevalence is 8.4% [21]. In order to prioritise confidentiality and create a comfortable environment for WWDs to share their experiences, interviews were conducted either at their residences or at the organisations they were affiliated with.

Sampling can be described as the procedure by which research participants or data sources are chosen to acquire data for addressing a study’s objectives [18]. Although sampling methods can be categorized into two main groups, probabilistic and non-probabilistic, this research employed non-probabilistic sampling. The target population for this study was WWDs who were currently pregnant or had been pregnant in the previous five years.

The researcher requested the KZN Department of Social Development (DSD) to provide a list of the names and addresses of WWDs residing in eThekwini district. The KZN DSD provided the researcher with a list of organisations that have WWDs. These women were at second trimester of their pregnancy or had been pregnant in the past five years. As this is a qualitative study, non-probability sampling was used in which the researcher purposively selected information-rich cases from which to collect data for the study [22]. In the current study, criterion sampling was utilised to select women with visual, hearing and physical impairments and those who were at second trimester of pregnancy or had been pregnant in the past five years [22,23]. Though some research indicates that in five years, fifty percent of one’s memory is lost, other studies have indicated that childbirth experiences have a powerful effect on women with the potential for a permanent or long-term positive or negative impact [24,25]. In addition, pregnancy and childbirth experiences are emotionally arousing experiences, and psychological studies have found that emotionally arousing experiences are more accurately and vividly remembered [26]. Therefore, in this research, women with disabilities who were pregnant or had had children in the past five years were considered.

A minimum of 12 WWDs were selected. Of these 12, four were women who were visually impaired, four had hearing impairments and four had physically impaired. As this was a qualitative study, the sample included only 12 participants due to logistical and financial reasons [27]. However, data collection continued until a point when no new information could be obtained from the WWDs. This sampling strategy enabled the researcher to account for the impact of the women’s different impairments on their access to maternal health services. In addition, this sampling strategy enabled the researcher to include WWDs who were currently pregnant and those who had been pregnant in the past five years. The researcher visited these women at their homes to request that they participate in the study. Necessary provisions for communicating with women with different impairments were put in place. For instance, in cases of women with hearing impairments, the researcher engaged a sign language interpreter.

Inclusion and exclusion criteria define who can be included and who is excluded in a study. The inclusion criteria serve to define the study’s population consistently, reliably, uniformly and objectively, while the exclusion criteria encompass factors or traits that render individuals unsuitable for participation in the study [28]. In this study, the inclusion criteria were as follows: WWDs (women with a physical or mobility impairment, or women with a sensory impairment such as impaired vision or impaired hearing) who sought public maternal healthcare services (antenatal, perinatal and immediate postpartum), WWDs who were pregnant in their first and second trimester, WWDs who had had a child in the past five years, WWDs residing in KZN and women aged 18 to 45 as this age range falls within the reproductive age bracket. The exclusion criteria included the following: women with cognitive impairments as they would not be able to give consent to participate in the study, WWDs who had never been pregnant, WWDs who were pregnant but were in their third trimester, WWDs who had never had a child or had had a child more than five years ago and WWDs who were less than 18 years old or more than 45 years old.

An interview is described as an extensive dialogue between individuals, aiming to acquire comprehensive insights into a specific topic or subject, enabling the interpretation of phenomena [25]. In this study, in-depth interviews were employed to collect insights into the experiences of WWDs concerning their access to maternal healthcare services. This data collection method facilitated a profound understanding of the phenomena being investigated by allowing for the clarification of vague responses and a deeper exploration of individuals’ perspectives on the discussed topics [25]. Ethical approval was obtained from the Durban University of Technology Institutional Research Ethics Council (IREC).

Since the researcher was not fluent in isiZulu, interviews were conducted by the researcher with the help of a research assistant, who was a Master’s student, understood the research methodology and was fluent in both English and isiZulu. Prior to the interviews, the participants were guided through the informed consent form. After reading the form’s content, they proceeded to sign it. After the consent process, the participants were taken through the interview guide, which encompassed inquiries pertaining to the primary research objectives. However, the interview guide was not used as a rigid structure. When necessary, the interviewer asked follow-up questions for clarification even if they were not included in the interview guide. If issues that were not addressed in the interview guide kept coming up repeatedly during the interviews, the interview was amended to include questions around these issues. The interviews were undertaken until data saturation point was reached. Data saturation is when no new information is obtained from interviews [29]. Interviews were conducted in the home or organisations of the WWDs.

Permission was sought from the participants to voice record the interview discussions. Each interview session took approximately 45 min to an hour to allow for a detailed discussion of the issues. The researcher took some notes during each interview to act as a backup in case the tape recording did not work. After each interview, the researcher compiled some notes on accessibility issues that were raised. After interviewing all the WWDs, the interviews were transcribed verbatim. However, the names of the participants were not included in the transcriptions to ensure confidentiality. Interviews that were undertaken in isiZulu were transcribed into the language used during the interview. These transcriptions were then given to a professional translator who holds a Master’s degree in Translation Studies to translate them into English. The second and third authors validated the translations.

The data was then analysed using the framework analysis. A framework analysis was utilised to analyse qualitative data. The framework analysis comprises five stages, which encompass familiarisation, identifying a thematic framework, indexing, charting and mapping interpretation [30]. The researchers used the process of transcription to start becoming familiarised with the data. The researchers continued listening to the audio recordings as well as studying the research notes to get a deeper understanding of the collected data. This process also aided the interpretation of the data and identification of the thematic framework. During the indexing process, the researchers identified portions of the data that corresponded with certain themes. Lastly, during the mapping and interpretation phase, the researchers analysed the key characteristics as laid out in the charts on matrices.

## 3. Results

To describe the experiences of WWDs in accessing maternal healthcare services, the authors conducted in-depth interviews with 12 WWDs. These WWDs were identified utilising the criterion sampling strategy. Of the 12 WWDs, four had physical impairments, with one having both physical and visual impairments. The participants also included four women with hearing impairments. Four of the participants had visual impairments. Of the four women with visual impairments, one woman also had albinism. The women were aged from 18 to 45 years. Six were aged between 18 and 25 years, four between 25 and 35 years, and two women between 35 and 45 years. All of the women were African and not employed. One of the women was enrolled as an Information Technology student at the eDeaf Association in Durban. Amongst the women, four were married while the other eight were not married. In terms of education, one woman did not have any formal education, one had a primary education, six had a secondary education and four had a tertiary education. The South African health system is organised into four levels of care. The first level for the provision of maternal healthcare services is at community healthcare centres and district hospitals. The second level includes regional hospitals which receive referrals from clinics and district hospitals. The third level is tertiary hospitals which receive referrals from regional hospitals. The tertiary hospitals refer the people that they cannot help to a national hospital, which is the fourth level of care. The included women had accessed maternal healthcare services from one of the levels of care in the eThekwini District. The eThekwini district was selected as it has facilities for all four levels of care (Table 1).

This study aimed to explore the factors that impact access to public maternal healthcare services for WWDs. The specific research question answered by this study was the following: what are the perceptions of WWDs with regard to their access to maternal healthcare services? The WWDs explained that they experienced numerous challenges when accessing maternal healthcare services. These challenges can be classified into three categories, which are (a) systemic factors, (b) structural factors and (c) personal factors. Some of their experiences varied depending on the type of impairment the WWDs had.

The systemic factors included the following: (a) information on and the communication needs of WWDs; (b) the knowledge and competency of healthcare workers to handle WWDs; and (c) the referral system for women with disabilities. Women with hearing impairments and those with visual impairments predominantly encountered challenges in having their information and communication needs met as individuals with disabilities. The subthemes that emerged were the following: (a) the lack of training in sign language among healthcare workers; (b) the lack of sign language interpreters; and (c) the lack of information in accessible formats.

### 3.1. Lack of Training in Sign Language Amongst Healthcare Workers

The lack of training among healthcare workers resulted in challenges in communication between healthcare workers and WWDs with hearing impairments. The excerpts provided below suggest that the absence of sign language training among healthcare professionals creates a source of frustration amongst WWDs when it comes to accessing maternal services.

*“Healthcare workers need to be able to communicate with the deaf because it’s very difficult to communicate with them because it’s very tiring to always arrive and have to communicate using a pen and paper. In government hospitals maybe if they can train nurses in South African Sign Language that would ease the communication problem”*.

(WWD2: 24 years).

*“They need to teach nurses sign language, or we can teach them sign language—maybe the basics such as “hello””*.

(WWD3. 33 years).

### 3.2. Lack of Sign Language Interpreters

The participants also indicated that a lack of sign language interpreters acts as a barrier to their access to maternal services. Sign language interpreters would make communication easier between healthcare workers and women with hearing impairments. This is explained in the excerpts below:

*“We cannot communicate with the deaf, you must come with the person…maybe your boyfriend or your sister so that they could interpret for you…that is what the nurses said”*.

(WWD3: 33 years).

*“Please, please, please, we need interpreters…there’re no interpreters and also in clinics there’re no interpreters there…but hoping that everything will be improved”*.

(WWD2: 24 years).

The lack of sign language interpreters negatively impacted the WWDs i-n accessing antenatal care, as illustrated in the excerpt below:

*“I went to the antenatal classes once and there was no interpreter. I could not hear anything, so I decided not to go as I did not gain anything from the classes”*.

(WWD3: 33 years).

Their challenges in communication were even greater during labour. The women indicated that they just had to follow the signs that the healthcare workers improvised and look at the facial expressions of the healthcare workers.

However, women with hearing impairments indicated that communication with healthcare workers was much better if they were able to read and write and when they had family and friends to interpret for them. This is confirmed in the following excerpts:

*“There was a communication barrier, but I was able to communicate with a doctor using a pen and paper”*.

(WWD2: 24 years).

*“The communication with the nurses was really difficult and I asked my sister to come with [me] to the clinic…”*.

(WWD3: 33 years).

### 3.3. Lack of Information in Accessible Formats

The women with hearing impairments expressed concerns of information being provided in inaccessible formats. One woman with a visual impairment had the following to say:

*“At the antenatal class, they had pictures and used actions to explain to us how we breastfeed our kids. I could not see anything. It did not make sense for me to attend further”*.

(WWD9: 38 years).

Another systemic factor alluded to was the knowledge and competence of healthcare workers. The WWDs explained that they had to contend with healthcare workers who did not understand their needs. The provided excerpts below illustrate healthcare workers’ limited understanding of the needs of individuals with disabilities.

*“It surprises the healthcare workers that we get pregnant. It’s like we do not have functional reproductive systems. They need to be educated that we too have functional reproductive systems, and it is normal for us to be pregnant as we are women too”*.

(WWD9: 38 years).

*“The healthcare workers need to be sensitised on how to be disability-inclusive in the provision of maternal healthcare services… They should be provided with regular training because if the training is one-off they forget”*.

(WWD.7: 46 years).

### 3.4. Referral System for Women with Disabilities

The WWDs also identified the referral system for pregnant WWDs as one of the systemic factors that impact their access to maternal healthcare services. Our study found that WWDs were more likely to be referred to higher levels of care as illustrated in the excerpt below:

*“The nurses from the clinic said I should go to hospital X because they do not know what caused me to stroke even though the stroke happened when I was 5 years old”*.

(WWD1: 27 years).

When WWDs are referred to higher levels of care, they have to travel longer distances from their places of residence to the facility that they are referred to.

### 3.5. Structural

Structural factors included: (a) the infrastructural design and layout, (b) examination beds, (c) sanitary facilities and (d) disability-friendly scales. Women with visual impairments and women with physical impairments had to contend with navigating facilities and equipment that are not disability-friendly.

### 3.6. Infrastructural Design

Some of the facilities had narrow passages as well as narrow doors and did not have ramps. One woman with a visual impairment shared her frustrations below:

*“The hospital has so many stairs and it is difficult for me as I am visually impaired. My sister helped me to move from one place to another”*.

(WWD7: 35 years).

A woman with a physical impairment who uses a wheelchair visited a community healthcare centre and explained how the healthcare worker had to consult her while she was in the corridor:

*“I had a problem, Um, my wheelchair, it could not fit into the consultation room. The nurse had to come and see me in the passage, and I was then told to go to hospital X for my next visit”*.

(WWD12: 38 years).

Women with physical impairments also had challenges with unadjustable beds, bathrooms that did not have rails and a lack of disability-friendly scales. The excerpts below reflect the experiences of the WWDs with physical impairments when they faced unadjustable beds and bathrooms without rails.

*“Because I have one leg, it was difficult for me to climb onto the bed. I had to be assisted by the nurses… they had to physically carry me”*.

(WWD8: 34 years).

*“My partner had to assist the hospital sister to carry me onto the examination bed. It was difficult for me to get onto the bed on my own”*.

(WWD1: 27 years).

*“They did not weigh me. They would do the other check-ups but because I could not stand on the scale, I was never weighed”*.

(WWD12: 38 years).

### 3.7. Personal Factors

Personal factors that were alluded to by the WWDs included (a) healthcare worker attitudes towards the WWDs and (b) the availability of a companion.

Healthcare worker attitudes toward WWDs

Negative staff attitudes were cited as having a negative impact on their access to maternal healthcare services. This is outlined in the excerpts below:

*“Because at Hospital X I felt that they did not understand that I too can have babies and kept on asking that and mocking me about it, I then decided to go to Hospital Y for my second baby. However, at Hospital Y still, there were nurses who were saying I love men too much, that is why I keep getting pregnant”*.

(WWD7: 35 years).

*“In hospital where I delivered my baby, they ignored me and said “ you will deliver on your own and keep on crying like that” and finally I had to have an operation because I had felt severe labour pains and the baby was tired…Because my first born almost lost his life at hospital A because of their negligence, I then decided to change the address in order for me to deliver at X”*.

(WWD5: 35 years).

*“It was a very difficult time; some of the hospital nurses would just ignore. I felt the nurses were ignoring me maybe because I’m deaf and they were attending the hearing patients…They gave the hearing women fair treatment, but they were ignoring me because of my deafness”*.

(WWD3: 33 years).

### 3.8. Availability of a Companion

Most WWDs require someone to accompany them during hospital visits. Women with hearing impairments require someone to interpret for them, those with visual impairments require someone to assist them with the navigation of facilities and those with physical impairments need assistance manoeuvring around facilities. The need for a companion increases the cost of services, as illustrated in the excerpt below:

*“They said, “we cannot communicate with the deaf, you must come with the person…maybe your boyfriend or your sister so that they could interpret for you–that was the nurses”*.

(WWD2: 24 years).

In the excerpt below, one woman explained how she had additional costs as she needs to always have someone to assist her during hospital visits:

*“As I am blind and have one leg when I need to go to the clinic for post-natal care, I need someone to accompany me to carry the baby for me. This means I need transport fees for two people”*.

(WWD7: 38 years).

The excepts revealed that the WWDs experience numerous challenges in accessing maternal healthcare services. These challenges include communication, inaccessible buildings and equipment, and the negative attitudes of healthcare workers. The results also indicate that having a companion when seeking services is a facilitator for access. These findings are interpreted in the discussion section.

## 4. Discussion

The results presented revealed the barriers and facilitators of access to healthcare services that are experienced by the WWDs. This section discusses the findings and the implications of these findings for access to maternal healthcare services for WWDs.

### 4.1. Barriers of Access to Maternal Healthcare Services for WWDs

The findings revealed that the WWDs experienced numerous barriers of access to maternal healthcare services. These barriers include communication challenges, the negative attitudes of healthcare workers, the referral of WWDs to higher levels of care, additional costs, and infrastructural design and layout.

### 4.2. Communication Challenges

This study revealed that healthcare workers cannot communicate in sign language resulting in the need for sign language interpreters. Sign language interpreters are important in ensuring that healthcare workers and women with hearing impairments can interact effectively. This study found that the facilities did not have sign language interpreters. The lack of sign language interpreters led to the women with hearing impairments relying on their relatives to interpret their sign language for healthcare workers. The lack of sign language interpreters and the issue of relying on relatives to be interpreters are also reported in other studies [31,32]. Ineffective communication between healthcare providers and women with hearing impairments can lead to delays in diagnosis, misdiagnoses or incorrect assessments, ultimately resulting in poorer health outcomes [33,34].

### 4.3. Negative Attitudes of Healthcare Workers

Some of the WWDs reported being treated with disrespect by healthcare workers. The WWDs noted that not all healthcare workers were disrespectful. The disrespect was displayed in the form of yelling, ignoring calls from the WWDs and indirectly telling the WWDs that they should not get pregnant. Such kinds of abuse have also been documented in studies for women without disabilities [35]. Such abuse dissuades WWDs from visiting healthcare facilities early. Although disrespectful treatment has been recorded in studies for women without disabilities, it is important to note that having a disability worsens one’s situation. These negative attitudes may be due to a lack of training and knowledge amongst the healthcare workers as well as assumptions on what WWDs can and cannot do. These findings are consistent with other studies conducted in South Africa by Mavuso and Maharaj and Gichane et al. [36,37] as well as in other parts of the world [8,38,39]. These studies report that the social beliefs of healthcare workers, who perceive WWDs as asexual and not capable of mothering babies, lead to WWDs feeling disrespected. When WWDs feel disrespected, they avoid facilities and deliver at home [40]. In some instances, WWDs seek services from other facilities that may be further away from their places of residence, increasing their travel costs to seek services.

### 4.4. Infrastructural Design and Layout

This research revealed that significant efforts are required to make maternal health institutions more accommodating for individuals with disabilities. Many of these facilities had cramped corridors that posed challenges for women with physical or visual impairments to move through, and in certain instances, ramps were lacking. The elevators in some facilities did not work and this situation was exacerbated by load shedding. These findings are in line with the findings of the study conducted in Durban by Mavuso and Maharaj [37] and other studies in low- and middle-income countries [41,42,43] which outline that most maternal health facilities are not designed to suit the needs of WWDs. The situation of WWDs is worsened by unadjustable equipment and sanitary facilities that are not disability-friendly. These findings are consistent with findings from other African countries [44,45].

### 4.5. Additional Costs

Even though maternal healthcare services are free, this study found that WWDs incur additional costs as they must travel to higher levels of care that are not in their local areas as well as have a companion to travel with. These additional costs are a burden to WWDs as the study revealed that most of the WWDs included are not employed. A lack of employment leads to individuals with disabilities having less financial means available to them. However, these WWDs are regarded as high-risk and hence are referred to higher levels of care. In most instances, their referrals happen when they are not emergency cases, and hence, they are required to transport themselves to the higher levels of care that are not within their locality. This increases the distance they travel from home to seek health services, which in turn increases their transport costs. Their transport costs are worsened by the fact that WWDs need to be accompanied by a family member or partner to facilities. This makes their transport costs higher than those for pregnant women without disabilities. Other studies have also reported that WWDs incur additional transport costs as they have to be accompanied and are also referred to higher levels of care [44,45]. While health facilities are accessible with public transport, in some areas, WWDs have difficulties in accessing public transport as it can be a bit far from their homes. Mavuso and Maharaj examined access to sexual reproductive health services for people with disabilities and also found that the referral of WWDs to higher levels of care increases the distance between the WWDs and the facilities where they have to go to access services [37]. 

### 4.6. Facilitators of Access to Maternal Healthcare Services for WWDs

This study revealed that the availability of a companion during health service visits was a facilitator of access to maternal health services. Companions assist with sign language interpretation for women with hearing impairments, navigate the facility for women with physical and visual impairments and carry women with physical impairments onto and off of high fixed beds. These findings are consistent with those from other studies which also found that family members assist with interpreting sign language and navigating health facilities [31,37,45]. However, a study by [31] found that companions are restricted to outpatient services during visiting hours, and women with hearing impairments do not have an interpreter at all other times. This caused challenges in communication between healthcare workers and women with hearing impairments during their delivery.

## 5. Conclusions

A few of the WWDs perceived the maternal healthcare services to be of good quality. The visually impaired raised concerns about narrow passages that are difficult to navigate and also information that is not accessible in braille. Women with hearing impairments raised concerns of challenges in communication as well as negative attitudes, rudeness and being ignored when asking for help. Physically impaired women raised issues regarding inaccessible buildings, narrow passages and small consultation rooms as well as beds that are not adjustable. Women with visual impairments faced challenges with information in inaccessible formats and having to navigate facilities with narrow passages and stairs. 

Research on WWDs’ access to maternal healthcare services is still very scarce. There is a need for more extensive research on WWDs’ access to maternal healthcare services and broadening our understanding of the factors that impact access for pregnant WWDs in order to develop disability-friendly maternal healthcare services. The World Health Organisation (WHO) recommended that information is obtained on the health needs of people with disabilities in order to make facilities disability-friendly [46]. As a result, there is a need for additional research that explores maternal healthcare needs and the utilisation of maternal healthcare services according to the type of impairment. Furthermore, our study revealed that healthcare workers’ knowledge, attitudes and beliefs impact access to maternal healthcare services. There is a need for research that explores the knowledge, attitudes and beliefs of maternal healthcare providers regarding maternal healthcare for WWDs.

In addition, the WHO recommends training healthcare workers on the provision of disability-inclusive services [46,47]. Maternal healthcare workers could be provided with in-service training. A Department of Health (DoH) task team to handle training could be established; this task team would provide a workshop for trainers. This task team would then be responsible for training healthcare workers from different districts. The district officials would then be responsible for training the maternal service providers of different facilities. As this is in-service training and a tool kit and training manual are available, this model would be cost-effective. Its challenges may include having enough time for the training of maternal service providers. This could be negotiated with district management teams.

Policymakers need to design policies that ensure the mainstreaming of WWDs in maternal healthcare services as recommended by the WHO [46]. These policies should include renovating maternal healthcare facilities so that they can accommodate WWDs without any challenges. In addition, policies should incorporate the equipping of maternal healthcare facilities with disability-friendly equipment and assistive devices. This may take a long time due to financial constraints in the economy in general and the public health sector in particular. Nevertheless, the suggested changes can be implemented utilising an incremental approach whereby a few facilities per district can be selected to be fully resourced and designed to suit the needs of pregnant WWDs. In addition, the government may partner with the corporate world to improve the existing infrastructure. The government can incentivise the corporate world by formulating fiscal policies that will ensure that corporate stakeholders receive high tax refunds. A partnership between the government and the corporate world should also address issues of the shortage of healthcare workers in the public maternal healthcare system. If this recommendation is implemented successfully, WWDs will easily access public maternal health facilities, resulting in a reduction in maternal mortality, which is one of the challenges facing low- and middle-income countries including South Africa.

## Figures and Tables

**Table 1:** Demographic profile of women with disabilities.

Participant No.	Age (Years)			Race	Marital Status		Educational Level				Employment Status	Type of Impairment		
	18–25	25–35	35–45		Y	N	None	P	S	T		Visual	Physical	Hearing
WWD.1		X		African		X	X				Not employed		X	
WWD.2	X			African		X				X	Student			X
WWD.3			X	African	X					X	Not employed			X
WWD.4		X		African	X					X	Student			X
WWD.5		X		African	X				X		Not employed			X
WWD.6		X		African	X				X		Employed		X	
WWD.7			X	African		X			X		Not Employed	X		
WWD.8		X		African		X			X		Not employed	X	X	
WWD.9			X	African		X				X	Employed	X		
WWD.10	X			African		X			X		Not Employed	X		
WWD.11		X		African		X			X		Not employed		X	
WWD.12			X	African		X		X			Not Employed		X	

WWD = Woman with disability, Y = Yes, N = No, P = Primary, S = Secondary, and T = Tertiary.

## Data Availability

The data presented in this study are available upon request from the corresponding author. The data are not publicly available due to privacy restrictions.
